# Evaluation of the Mechanism of the Gold Cluster Growth during Heating of the Composite Gold-Polytetrafluoroethylene Thin Film

**DOI:** 10.3390/nano2040366

**Published:** 2012-11-07

**Authors:** Konstantin Grytsenko, Valeri Lozovski, Galyna Strilchuk, Sigurd Schrader

**Affiliations:** 1 Institute of Semiconductor Physics, National Academy of Sciences of Ukraine, Nauki ave. 41, Kyiv 03028, Ukraine; Email: d.grytsenko@gmail.com (K.G.); v.lozovsk@gmail.com (V.L.); 2 Institute of High Technologies, National T.Shevchenko University of Kyiv, Volodymyrska str. 64, Kyiv 01001, Ukraine; 3 University of Applied Sciences, Wildau, Bahnhofstrasse 15745, Germany; Email: sigurd.schrader@th-wildau.de

**Keywords:** nanocomposite, gold, polytetrafluoroethylene, optical spectroscopy, microscopy, thin film

## Abstract

Nanocomposite films consisting of gold inclusions in the polytetrafluoroethylene (PTFE) matrix were obtained by thermal vacuum deposition. Annealing of the obtained films with different temperatures was used to measure varying of film morphologies. The dependence of optical properties of the films on their morphology was studied. It was established that absorption and profile of the nanocomposite film obtained by thermal vacuum deposition can be changed with annealing owing to the fact that different annealing temperatures lead to different average particle sizes. A method to calculate the optical properties of nanocomposite thin films with inclusions of different sizes was proposed. Thus, comparison of experimental optical spectra with the spectra obtained during the simulation enables estimating average sizes of inclusions. The calculations give the possibility of understanding morphological changes in the structures.

## 1. Introduction

Nanoparticles of noble metals are widely studied on account of their fundamental and practical applications in virtually all fields of the applied sciences. Due to their excellent stability and wide range of unusual electronic properties [1,2,3] they are used for photonics [4], plasmonics [5], waveguides [6], sensors [7,8], photovoltaics [9], biology and medicine [10,11], heterogeneous catalysis [12], *etc.* Different wet and gas phase deposition methods are used in order to obtain Au nanoclusters inside different types of matrixes. These methods are reviewed in [13,14,15]. Particularly, evaporation of Au and polytetrafluoroethylene (PTFE) in a vacuum with vapor co-condensation on cold surface was reported in [15]. The PTFE prevents growth of continuous Au film, therefore nanocomposite film is formed. Due to practical necessities, nanocomposites are often made as thin films and have optical properties which differ from the properties of bulk objects made of the same material [16]. The main reason for these differences lies in the presence of a strongly inhomogeneous local field at the interfaces [17]. For example, these interface effects are the reason for a depolarization phenomenon [18]. There are numerous works focused on the study of the optical properties of small particles or small particle systems [19,20,21]. There are some papers concerning the optical properties of thin and ultrathin films [18,22,23,24,25,26,27]. On the other hand, the three-component nanocomposite thin films attract attention of technologists and physicists due to the wide potential of the varying of its physical properties. A solution for technological problems leads to the need of morphological estimation of deposited film using nondestructive methods. Namely, one needs to know the concentration and shape of the inclusions, its distributions inside the film, *etc.* In the general case, these data can be obtained from rather complicated measurements. For example, the shape, dimensions and distribution of inclusions can be determined by the electronic microscopy. The surface inhomogeneities can be visualized by a scanning optical microscopy, *etc.* In the main cases, these measurements cannot be made *in situ*. Therefore, there is a need for a rapid experimental method supplemented by a theoretical modeling. This method allows evaluating the size, shape, and shape distribution of the inclusions inside the nanocomposite film. The extensive methods for these purposes are the optical methods. Measurement of absorption spectra is the simplest and, at the same time, the most informative optical method. There is an increasing possibility of the optical absorption spectroscopy use for the study of the three-component nanocomposite film structure. Moreover, measurement of absorption spectra of nanocomposite films can be carried out *in situ* during the film deposition [14]. Then, having an appropriate model for the description of the measured optical absorption spectra, one can make some conclusions about the film structure. As a result, the main goal of the present work is to propose the modeling method of absorption properties of three-component nanocomposite thin film.

The question is: what does a thin (or utra-thin) film mean in the context of the problem?

There are at least two aspects of the question to be pointed out. The first aspect is connected to the problem of boundary conditions. The second aspect is associated with taking into account the inhomogeneity effects of the local field inside the film. It is known that standard (Maxwell) boundary conditions can be used when the transient layer length is the smallest length parameter [28]. In this case, the interface can be considered perfect and the term “boundary conditions” is well defined. When the transient layer length is roughly equal to the thickness of the film, the boundary conditions cannot be correctly defined. Obviously, in this case, the local field inhomogeneity will play an essential role in optical properties of the film.

To calculate optical absorption profiles, influences of the field inhomogeneities inside the thin films should be taken into account. These inhomogeneities are caused by surface and transient layers near interfaces. Obviously, thickness of transient layers for perfect surfaces is about a few interatomic distances. Thicknesses of transient layers may change in the wide ranges ~10–1000 nm for real samples. Thus, limitation of the thin film thickness cannot be well defined in the context of the problem. Nevertheless, the thin film is considered to be a film with the thickness of less or about the wavelength of absorbed light, when it can be supposed that an electromagnetic wave does not propagate along the thickness of the film (near-field regime). Both of discussed aspects—a boundary conditions ambiguity and a near-field regime of field propagation across the film thickness—can be solved in the framework of the “local field approach” [29]. This approach consists in calculating the local field inside the object by solution of the self-consistent equation. The adequate method of approach implementation can be based on the effective susceptibility concept. The main idea of this concept lies in the difference of linear response to the local and external fields. This idea enables us to write a solution of the self-consistent field in an analytical form. Indeed, taking into account that in the plane of the film the system is homogeneous, one can make Fourier transformation in the film plane, then one can transform to so-called **k***-z* (or Wail) representation [30]. In this representation of the equation of the self-consistent field (the Lippmann-Schwinger equation) it takes the form [17].

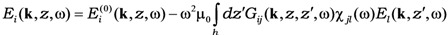
(1)
where the integration is over the thickness of the film, 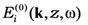 is a long-range external field acting to the film, 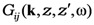 is the electrodynamic Green function (photon propagator) of the medium, in which the film is embedded, and 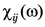 is the linear response to the local field, connecting the local current inside the film with the local field 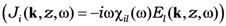. Introduction of effective susceptibility 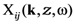 connecting the local current with external field 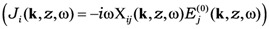 [17] enables us to write the solution of Equation (1) as
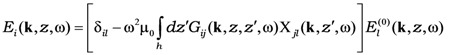
(2)


Structural changes of the films can be theoretically estimated using Equation (2). In particular, it needs to be taken into account the variation of the particle sizes. There are certain aspects that must be taken into account in the theoretical calculation. They are the morphological parameters of the film, such as a refractive index of the PTFE, density of the PTFE, and variation of the Au particle shape and size during the annealing.

The aim of this work is to develop methods of control of the Au cluster size and to affect optical properties of the nanoclusters ensemble.

## 2. Experimental Details

**Figure 1:**
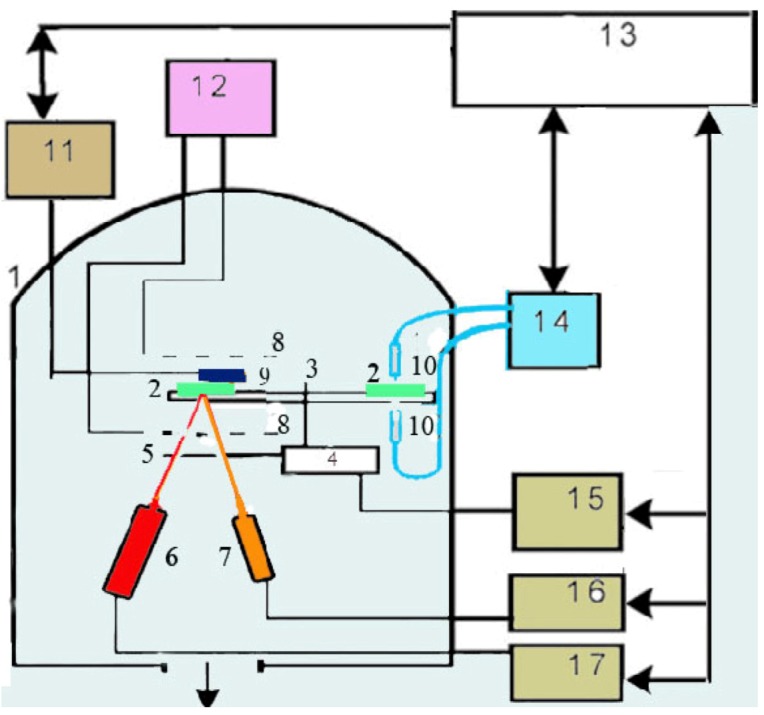
Setup of the deposition installation: (1) vacuum chamber; (2) substrates; (3) system and (4) motor for substrate rotation; (5) shutter; (6) evaporator-activator for polytetrafluoroethylene (PTFE); (7) evaporator for Au; (8) RF electrodes; (9) quartz crystals of Sigma card; (10) fibres; (11,15–17) controllers of quartz monitors, motor, PTFE and Au evaporators; (12) RF generator; (13) computer; (14) optical spectrometer StellarNet.

There are a number of methods for the preparation of metal-insulator nanocomposite films [8,10,11,12] which can be used for creating such films. Our films were made by thermal vacuum deposition. Films were deposited by using UVN-74 (USSR) industrial installation equipped with Pfeiffer vacuum pressure meter, Sigma quartz thickness monitor and optical spectrometer StellarNet (Figure 1). The computer receives signals from these devices, collects and analyzes the data *in situ.* A rotating glass disk was attached with Si, NaCl, glass and quartz slides, which were used as substrates. PTFE was evaporated with vapor activation by electron cloud. The heated tungsten boat was used for gold evaporation. Further details can be observed in [14,15]. Optical properties of the obtained films were studied by measuring optical transmission spectra. The optical spectrometer equipped with fibers led-in vacuum chamber gives much diverse spectroscopic information on film growth processes *in situ*. The sample delivery system expands the set of sample operations at the time of film deposition and allows depositing uniform or wedge-like by thickness films when needed. Water-cooled walls of each evaporator shadow the thermal radiation and prevent samples from heating, which is significant for the condensation of volatile organic compounds. A computerized system carried out the process control.

Several sets with three samples were deposited, each set in one run; the samples for each set are distinguished only with filler concentration, caused by different shielding of atomic flow against substrate (three sets for Au: Samples 1–3). The structure of deposited film was studied by transmission electron microscope (TEM) JEM-100EX. Annealing of the films was made in the homemade oven equipped with an optical spectrometer Polytec and a computer installation for spectra recording *in situ*. The film morphology was studied by an atomic force microscope (AFM) Nanoscope IIIa Dimension 3000™ after annealing at room temperature in a tapping mode. This method allows the creation of a uniform distribution of Au in PTFE and to control and to modify growth processes *in situ*, in real time.

## 3. Experimental Results and Discussion

**Figure 2:**
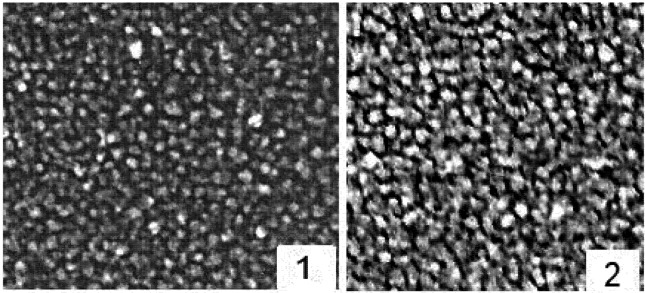
Transmission electron microscope (TEM) images of the Au-filled PTFE films. Au concentration increases from 1 to 2, from 5 to 21 vol %. Magnification 100,000.

The Au-filled PTFE films were deposited onto glass and quartz slides with Au concentration ranging from ~5 to 21 vol %. Depending on Au concentration, the film transmissions ranged from 70% to 30%. Deposition rate was in the range of 6–20 nm/min, and film thickness was in the range of 50–100 nm. The maximum of the absorption profile position for a final film was shifted from 510 to 550 nm with the increase of Au concentration. TEM investigations show that Au cluster size was defined by Au Figure concentration. The TEM images of the films with different Au concentration are presented in Figure 2.

Distribution of cluster sizes in the Au-PTFE film is in the range of 2–6 nm (5 vol % Au) and of 7–14 nm (21 vol % Au). A PTFE refractive index value was defined as 1.33. Electron diffractions patterns give a typical Au crystal structure. Clusters are somewhat spheroidal in form, but not completely spherical. Although, in order to achieve a high degree of accuracy, one should suppose that particles are of ellipsoidal shapes. A higher Au concentration is characterized with a bigger cluster size. At the higher concentration clusters, aggregation is presented in images. PTFE matrix prevents formation of smooth Au film, but also the Au clusters keep formation of the PTFE phase in thermodynamically disequilibrium conditions. It is amorphous. The red shift of the plasmon band maximum with the increasing Au concentration is due to both Au nanocluster size increases and their interaction. The light absorption profile maximum and shape were changed depending on Au concentration during the annealing. Each sample was annealed to the point where its characteristics do not change (according to observations of spectra).

**Figure 3:**
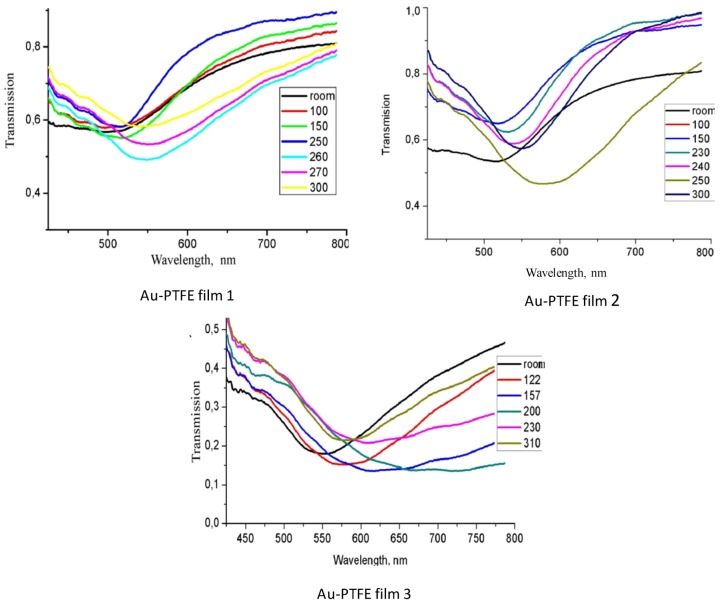
Optical transmission spectra of the Au + PTFE films recorded during the annealing. Au concentration is increased from 1 to 3 (from about 5 to 21 vol %).

The optical transmission spectra of the films recorded during the annealing are presented in Figure 3. In the films with low Au concentration, the band maximum was shifted from about 510 to 560 nm in temperature range of annealing from 100 to 260 °C, its absorption was decreased at 120 °C at first, but later was increased. At a further temperature increase to 300 °C, the plasmon band in the films with the smallest Au concentration was shifted a little more, and its absorption was decreased. In the film with the higher Au concentration, the plasmon band was shifted in the same in the 100 to 260 °C temperature range, but with further heating, the plasmon band was shifted back to the blue region. In the films with maximum Au concentration, the absorption maximum was shifted during heating and its band width was widened. The annealing of the Au-PTFE films deposited with additional plasma activation [31] showed that their transformation starts at about 200 °C. Therefore, a cross-linked PTFE matrix resulted in more stable film.

**Figure 4:**
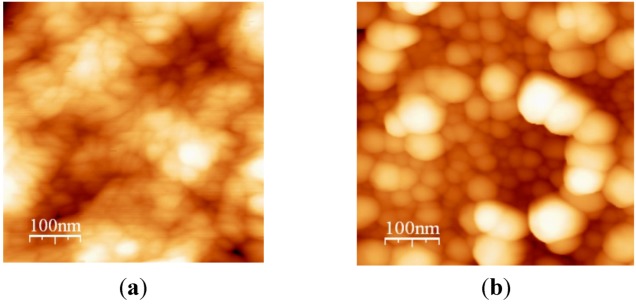
AFM image of Au-PTFE film 1 (**a**) T = 150 °C, size = 30–40 nm; (**b**) T = 220 °C, size = 60–80 nm.

**Figure 5:**
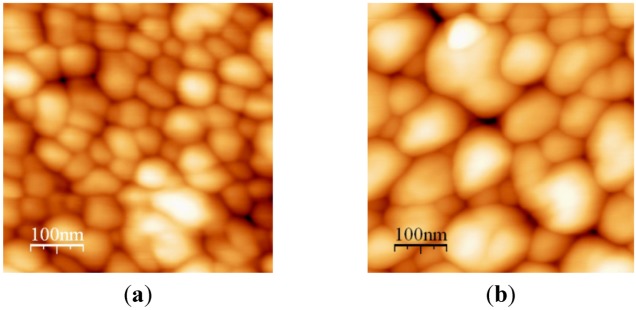
AFM image of Au-PTFE film 2 (**a**) T = 150 °C, size = 40–60 nm; (**b**) T = 220 °C, size = 70–100 nm.

**Figure 6:**
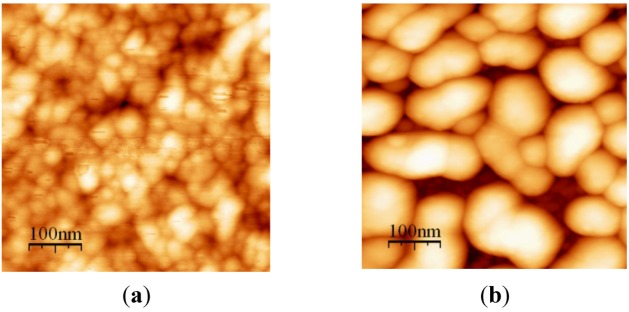
AFM image of Au-PTFE film 3 (**a**) T = 150 °C, size = 20–40 nm; (**b**) T = 220 °C, size = 80–110 nm.

The surface morphology of the film annealed at different temperatures is presented in Figure 4, Figure 5, Figure 6. At an elevated temperature, the Au diffusion rate increases, and the motion of the PTFE macromolecules increases, as well. The mean size of the Au clusters was increased at the first stage of heating by surface diffusion from small clusters to larger ones. The distance between clusters was increased. The PTFE phase also aggregated into larger particles and enlightened the Au atoms diffusion. At high Au concentration, the quasi-films were formed at 240 °C with preferable interactions in the film plane. At higher temperatures, the Au cluster growth continued, which led to an increase of distance between Au clusters and the suppressing of effects related to intercluster interactions. Kinetics of the Au clusters ensemble transformation during the heating is not linearly related to Au concentration. The Au cluster size distribution and properties of the PTFE matrix influence the Au atoms diffusion and new cluster growth processes. It should be mentioned that transformations are carried out at significantly lower temperatures than those necessary for such transformations in the pure gold films [32].

AFM images include both Au and PTFE nanoclusters. There are still two phases present in the films annealed up to 300 °C. Phase segregation into larger clusters enhances the film porosity and thus the large free surface for Au atoms diffusion.

The higher Au concentration obviously causes the more red-shifted absorption peak for both deposited and annealed films. Therefore, controlling Au concentration and annealing temperature, we can control the Au cluster morphology and film optical properties.

## 4. Modeling

The main visible change in the morphology of the annealed films is observed in the sizes of particles. It is proposed to consider the electrodynamics of thin and ultrathin films in the frame of self-consistent (local) field concept [27,29,33]. This approach is based on the Green’s function method. One calculates absorption profiles via the dissipative function. Calculation of absorption profiles is performed in the framework of effective susceptibility approach [17]. As it was shown in the numerous papers (see, for example, References [17,27], which shows how the absorption profile, in the case of normal incidence light, can be calculated.).

AFM images include both Au and PTFE nanoclusters. There are still two phases present in the films annealed up to 300 °C. Phase segregation into larger clusters enhances the film porosity, therefore, the large free surface for Au atoms diffusion.
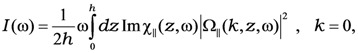
(3)
where

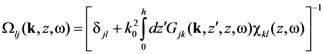
(4)
and subscribe || means in-plane components of the tensors 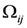 and 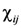.

The problem lies in defining the linear response to the local field. This problem can be solved when the optical properties of a nanocomposite (the linear response to the local field which is the characteristic of material) is defined within the framework of an effective medium theory [34,35,36,37,38]. One should point out that usage of the effective medium theory accompanies the requirement to linear dimensions of inclusions and its concentration. Namely, the characteristic linear dimension *a* of inclusions, the average distances between neighboring inclusions *l* and thickness of the film *h* should obey the next inequality.

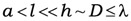
(5)
*D* is the characteristic length of transient layer at the film surface, and λ is the wavelength of probing radiation. As all technological conditions lead to rather spherically shaped particles, one can suppose that mainly different dimensions of inclusions are reasons causing different absorption profiles. Influence of particle sizes on optical properties of nanocomposite films can be estimated as it is proved in papers [38]. Namely, the influence of the dimension of spherically shaped inclusions on optical properties realizes via change of the collision rate due to diffusion scattering of electrons at a particle surface. Then the damping constant γ can be modified:

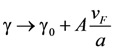
(6)
with 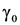 damping constant of material (Gold). Then, the calculation of the size influence can be carried out according to the equation:

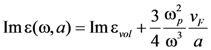
(7)

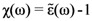
(8)
and 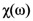 will play a role of the linear response on the local field of a three-component medium with two types of spherical inclusions of the same dimensions. Another type of a three-component system can be realized by compounding inclusions. It can be particles with a shell. One shall consider only such a type of another three-component system in this work. Then, according to [14,22,28], one considers the three-component composite consisting of coated particles embedded into a dielectric matrix. In order to calculate the effective dielectric function of this material, one will found the polarization per volume of the spherical particles with a shell:

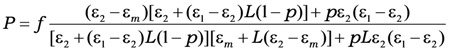
(9)
where *L* is the depolarization factor of the particles (for the case under consideration this is depolarization factor of a sphere—*L* = 1/3, a prolate ellipsoids correspond to *L* > 1/3 and an oblate *L* < 1/3), *ε_1_* is a dielectric constant of the material of the particle, *ε_2_* is a dielectric constant of material of a shell, ε*_m_* is a dielectric constant of a matrix, *p* is the volume part of the particle core (when *p*→*1* particle becomes without its shell), and *f* is the volume fraction of the inclusions.

Then, one should use the equation which connects the dielectric permittivity of effective medium (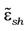) with polarization *P* of Lorentz sphere in the medium:

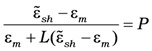
(10)


Comparing Equations (9) and (10) one can find the effective dielectric function of a three-component composite medium consisting of coated spherical particles embedded into dielectric matrix. Moreover, then, one can obtain the linear response on the local field:
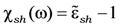
(11)


**Figure 7:**
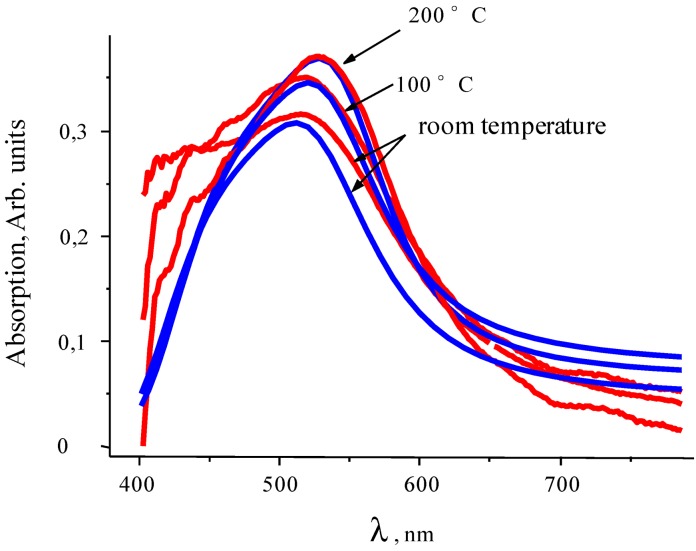
Extinction spectra of Au-PTFE film 2 during the annealing process. Blue lines correspond to modeling and red ones–to experiment.

**Figure 8:**
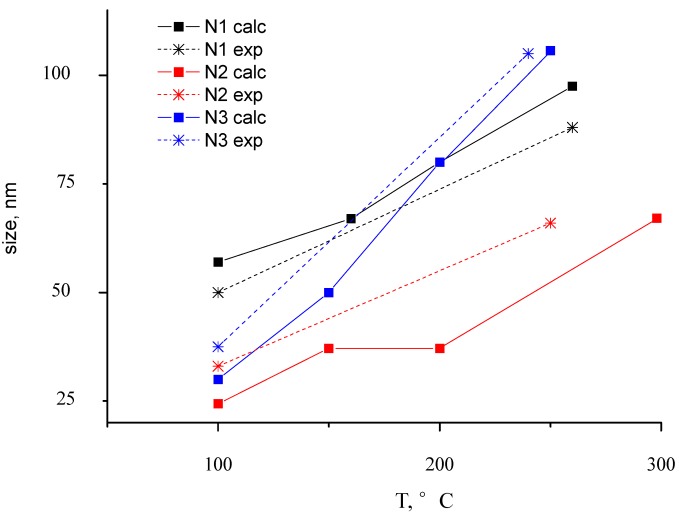
The dependence between particle size and annealing temperature. Experimental and theoretical dependence is shown for Au-PTFE film with different Au concentration (Samples 1–3).

Transmission spectra have been calculated in conformity with the absorption spectra. The absorption spectra of thin Au-PTFE film 2 calculated with different sizes of particles are represented in Figure 7. At this figure the experimental adsorption spectra are shown, as well. We can see that modeling absorption profiles show similar results with the ones obtained experimentally. As one can see, the different dimensions of inclusions lead to shifting of the spectra maximum. Corresponding to the shifting value, sizes of particles were represented during the annealing process (Figure 8).

Results have satisfied correlation with microscopic measurement. Dynamics of size changes with annealing is not linear. The tendency of changes in spectra repeats for all experimental cases.

In the curve N2 calc shown in Figure 8, the point that corresponds to the annealing temperature of 250 °C (it is marked as a square), because the particle size does not change so drastically, confirms the presumption, that spectra in this temperature range have the influence of low-annealing effects.

Careful attention is drawn to the variation of PTFE density with changing its annealing temperature. The shift in high frequency diapason is characterized by the choice of increasing PTFE density. Obviously, in this case, Au concentration is more than identified value.

Different sizes of particles can be achieved, depending on the annealing temperature. At the higher annealing temperature, we can get the large size of the particles. Annealing is also highly dependent on the initial parameters of the film.

## 5. Summary

The influence of annealing on the optical properties and morphology of the Gold-PTFE nanocomposite films was studied. The absorption band position and profile of the nanocomposite film obtained by thermal vacuum deposition can be changed with annealing. The Au concentration can be purposefully controlled during the film deposition. Then, during the film annealing, one can control the average of size and shape of the included particles. The shape and size of the particles inside the matrix were measured with the AFM and were recalculated by modeling. As a result, one obtained a new approach aimed at the control of morphology of obtained nanocomposite films by comparison of calculated optical properties in the framework of any model and experimental results.
